# Moisture contents and product quality prediction of Pu‐erh tea in sun‐drying process with image information and environmental parameters

**DOI:** 10.1002/fsn3.2699

**Published:** 2022-02-22

**Authors:** Cheng Chen, Wuyi Zhang, Zhiguo Shan, Chunhua Zhang, Tianwu Dong, Zhouqiang Feng, Chengkang Wang

**Affiliations:** ^1^ Faculty of Management and Economics Kunming University of Science and Technology Kunming China; ^2^ College of Agriculture and Forestry Pu'er University Pu'er China; ^3^ Pu'er Gaoshan Zuxiang Tea Garden Co., Ltd. Pu'er China

**Keywords:** deep learning, moisture content prediction, Pu‐erh tea, sensory quality evaluation, sun drying

## Abstract

In this study, moisture contents and product quality of Pu‐erh tea were predicted with deep learning‐based methods. Images were captured continuously in the sun‐drying process. Environmental parameters (EP) of air humidity, air temperature, global radiation, wind speed, and ultraviolet radiation were collected with a portable meteorological station. Sensory scores of aroma, flavor, liquor color, residue, and total scores were given by a trained panel. Convolutional neural network (CNN) and gated recurrent unit (GRU) models were constructed based on image information and EP, which were selected in advance using the neighborhood component analysis (NCA) algorithm. The evolved models based on deep‐learning methods achieved satisfactory results, with RMSE of 0.4332, 0.2669, 0.7508 (also with *R* ^2^ of .9997, .9882, .9986, with RPD of 53.5894, 13.1646, 26.3513) for moisture contents prediction in each batch of tea, tea at different sampling periods, the overall samples, respectively; and with RMSE of 0.291, 0.2815, 0.162, 0.1574, 0.3931 (also with *R* ^2^ of .9688, .9772, .9752, .9741, .8906, with RPD of 5.6073, 6.5912, 6.352, 6.1428, 4.0045) for final quality prediction of aroma, flavor, liquor color, residue, total score, respectively. By analyzing and comparing the RMSE values, the most significant environmental parameters (EP) were selected. The proposed combinations of different EP can also provide a valuable reference in the development of a new sun‐drying system.

## Graphical abstract

In this study, images were captured continuously in the sun‐drying process of Pu‐erh tea. Environmental parameters (EP) of air humidity, air temperature, global radiation, wind speed, and ultraviolet radiation were collected with a portable meteorological station. Moisture contents and product quality of Pu‐erh tea can be accurately predicted with deep learning‐based methods. The proposed combinations of different environmental parameters can also provide a valuable reference in developing a new sun‐drying system.

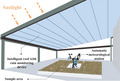

## 1 INTRODUCTION

Pu‐erh tea (PT) belongs to a particular tea class which originates from Yunnan Province in China. It is known to be beneficial for human health due to its antioxidative, anticancerogenic, and toxicity‐suppressing activities (Lv et al., 2013). The raw Pu‐erh tea (RPT) is usually processed through plucking, spreading, fixation, rolling, and sun drying. Among them, sun drying is the most crucial step and the basis for distinguishing RPT from other teas (Lv et al., 2013). In this step, moisture content must be controlled to an optimal level to achieve the best product quality. However, the moisture content was difficult to estimate instantly with traditional methods. Also, with the loss of water from the tea leaves, the total flavonoid, phenolic, vitamin C content, chlorophyll II, antioxidant activity, ascorbic acid equivalent are changing significantly (Chan et al., 2009; Roshanak et al., 2016). These changes are not only determined by the tea characters themselves but also related to the environmental parameters (EP) such as air temperature, air humidity, global radiation, light intensity, wind speed, and ultraviolet radiation. Hence, online monitoring of the EP and fast prediction of the moisture content are necessities in the PT industry.

Traditionally, during the sun‐drying process of PT, the estimation of moisture content was conducted by the tea makers with their eyes and hands, and the evaluation of product quality was upon the sensory scoring performed by a panel of trained tasters after sun drying. The estimation and evaluation accuracy was based on people's experience, mood, and mental state, which was not stable and consistent (Zhi et al., 2017). Recently, some sophisticated instruments, such as near‐infrared spectroscopy (Huang et al., 2021; Zhang et al., 2020), hyperspectral imaging (Wei et al., 2019), micro‐NIRS (Wang et al., 2021), electronic nose (Tudu et al., 2009), and electronic tongue (He et al., 2009), have been used for tea moisture and quality prediction. These methods can provide good results, but require professional knowledge and expensive equipment. Hence, a rapid and convenient evaluation method is still expected.

Computer vision (CV) is an engineering technology that combines electromagnetic sensing, mechanics, digital video, and image processing technology (Zareiforoush et al., 2015). Evidence has proved that CV is suitable for food quality evaluation (Wu & Sun, 2013), such as moisture detection of black tea in the withering process (An et al., 2020), prediction of moisture content for Congou black tea (Liang et al., 2018), rapid identification of tea quality (Xu et al., 2019), quality monitoring during black tea processing (Wang, Li, Liu, et al., 2021), determination of black tea's fermentation quality (Dong et al., 2018), evaluation of black tea fermentation degree (Jin et al., 2020), and identification of tea category (Zhang et al., 2016). The above studies mainly used color histogram, wavelet transforms, and gray‐level co‐occurrence matrix as image information extraction methods, and used support vector machines (SVM), multilayer perceptron (MLP), and radial basis function (RBF) neural network to fit the data for quality prediction. Although these methods can reduce the amount of calculation and improve the program's execution speed, they still have the disadvantage of accuracy decreasing in most situations. Recently, with the development of high‐performance computing (HPC) and Graphics processing unit (GPU), deep learning (DL) methods have become a promising approach in various food quality evaluations. Among these technologies, convolutional neural network (CNN) and Gated recurrent unit (GRU) combination has been successfully applied in tea leaf disease recognition (Chen et al., 2019; Hu et al., 2019; Hu et al., 2019), apple flower detection (Dias et al., 2018), fishery pond dissolved oxygen prediction (Li et al., 2021), and univariant time series forecasting (Saini et al., 2020). The increased computing ability on HPC and GPU allowed people to efficiently process high‐dimensional variables, which further promotes their practical applications.

Limited studies have reported the quantitative evaluation of PT moisture content and product quality in sun‐drying process using DL‐connected CV techniques. Thus, this study aimed to assess the feasibility of using the image and environmental information based on DL‐connected CV techniques for moisture content and product quality prediction. The specific objectives of this study were to:

1. use an industrial camera and a meteorological station to collect image information and EP during sun‐drying process;
2. establish prediction models based on the image information of tea leaves to predict its moisture content;
3. construct prediction models based on the image information and EP of sun‐dried tea to predict the final product quality; and
4. select the most influential EP by comparing the change rate of RMSE for optimization of the sun‐drying process of PT for future studies.

## 2 MATERIALS AND METHODS

### 2.1 Sample preparation

The fresh leaves of PT were collected from large‐leaf tea trees in Simao District, Pu‐erh City, Yunnan Province, China. Grade III samples were used in this experiment according to the Chinese national standard GB/T 22111‐2008: More than 50% of samples were one bud with two leaves or one bud with three leaves, and the others were buds or leaves with the same tenderness. A total of 100 batches of PT were used in the current study. The spreading, fixation, and rolling were all done by machines, and the processing conditions were set to be the same for all batches to reduce the impact of uncertain factors. During the sun‐drying process, sample tests (image capture and moisture content measurement) were performed every 2 h, and moisture detection was performed immediately after the images of leaves were captured to reduce the measurement errors. A total of 546 sample tests were conducted during the sun drying of the 100 batches of tea leaves. The EP of each batch of tea were collected for analysis. After the sun‐drying processes, the sensory scores of the tea products were given by a well‐trained panel.

### 2.2 Image information collection

A machine vision system in a separate room beside the drying shelter was used to capture the images of tea leaves during the drying process. The system consists of an industrial color camera (MV‐CE120‐10GC, HIKROBOT TECHNOLOGY CO., LTD., Hangzhou, China) with 8 million pixels and a C‐mount Varro‐focal lens (MVL‐MF0828M‐8MP, HIKROBOT TECHNOLOGY CO., LTD.), as well as a uniform light source which can be adjusted on an articulating arm boom. The uniform light emitted by the light source in the room can provide better tea leaf images without shadows. The interior wall of the room was painted white to achieve a uniform diffuse reflection. Image capture included three steps: (1) 15 ± 0.5 g of PT leaves in the sun‐drying process was placed in a glass vessel and spread uniformly, (2) the glass vessel was moved to a separate room and placed on a sampling platform in the machine vision system, and (3) the images of tea leaves were captured. The parameters of the machine vision system were optimized as follows: (1) The industrial camera's white balance was set to automatic mode to correctly display the color of tea and reduce the negative effect by illumination changes. (2) The aperture was adjusted to make the photo's brightness moderate so that the tea leaves could be clearly visible. (3) The working distance of the camera was fixed at 200 mm. After image capture, the raw digital image was saved as bmp files based on RGB color system.

In the whole sun‐drying process, the original images of tea leaves were collected every 2 h. It is worth noting that the last sampling intervals may be <2 h due to a possible early finishing of the drying process. The end time of the sun‐drying process was judged by the experts in the factory. Since the total drying duration required for different batches of tea was different, different numbers of images of 100, 100, 100, 89, 41, 16, and 100 were collected at the zeroth, the second, the fourth, the sixth, the eighth, the tenth, and the ending hour, respectively. A total of 546 images of all the above samples were used for moisture analysis, but only the images of the final products were used for product quality evaluation.

### 2.3 Moisture detection

In each sample test, the 300‐g PT leaves was collected and mixed evenly. Three grams was used for the moisture content measurement by a halogen moisture analyzer (AMTAST MB65, Amtast USA Inc.). Referencing the Chinese standards of GB/T 8304‐2013, the tea samples were placed in a drying vessel and heated to 120℃ for 1 h. The weight loss before and after drying was recorded. The ratio of the changed value to the original weight was the moisture content (wet based).

### 2.4 Monitoring of EP

**FIGURE 1:**
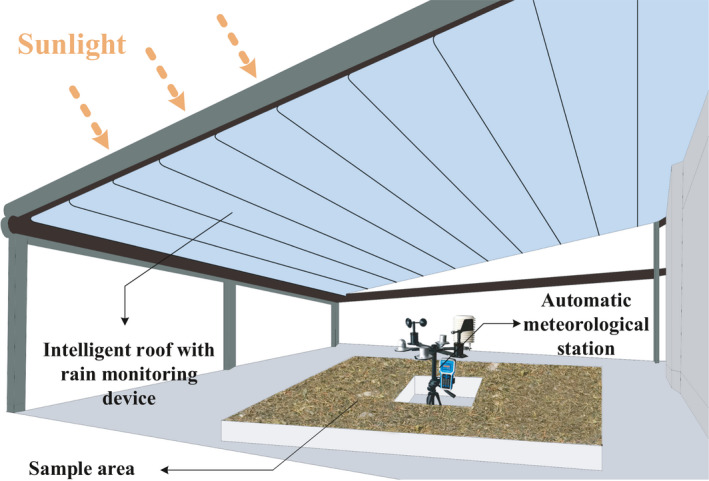
Drying shed with environmental parameter monitoring system

Pu‐erh tea production specifications (Lv et al., 2013) were followed for the selection of the experimental site. The experimental area was located in a tea factory. A drying shed was used in the experiment. To maximize sunshine duration in the daytime, the shed was finally placed on the top of a factory building. As illustrated in Figure 1, an intelligent plastic roof was installed on the shed, which can monitor air pressure and rainfall, and automatically close or open before and after rains. There are ditches around the shed to prevent rainwater from flowing into the experimental area. The shed roof was usually open and free of obstructions to ensure better ventilation, in case of no rain. An automatic meteorological station (RS‐QXZM‐M3‐Y‐4G, Shandong Renke Control Technology Co., Ltd.) was mounted in the center of the sample area to collect the EP, including air temperature (℃), air humidity (%RH), total radiation (W/m^2^), light intensity (lux), wind speed (m/s), and ultraviolet radiation (mW/cm^2^). The measurement error of the temperature sensor was ±0.4℃, and the humidity sensor error was ±2%RH. The range of measurement is 0–60℃ for the air temperature sensor and 1%–100%RH for the air humidity sensor.

In the meteorological station, global radiation was measured with a pyranometer, the measuring range of the pyranometer was 0–1800 W/m^2^, and the resolution was 1 W/m^2^. Furthermore, light intensity was measured by the light sensor. The light sensor is a light‐dependent resistor (LDR) that works based on the semiconductor photoelectric effect. The light sensor has a resistance that varies with ambient light intensity. By determining the corresponding relationship between resistance and illumination, the light intensity can be calculated. The light sensor can measure the light intensity of 0–200,000 lux with an error of no more than 7%.

The wind speed was measured with a sonic anemometer. The wind information was gathered when it passed through the spaces between the antennae. The sonic anemometers measured the time it takes for an ultrasonic pulse to move from one transducer to another. Compared with the cup and propeller anemometer, the sonic anemometer has fewer moving parts and less inertia, so the results were more accurate and reliable. The sonic anemometer can measure the wind speed of 0–60 m/s with an accuracy of 0.2 m/s.

Ultraviolet radiation was measured with an ultraviolet intensity meter in the station. The ultraviolet intensity meter consists of a silicon photocell and a microampere meter. The photovoltaic element converts light energy into electrical energy. The incident light passes through the metal film to reach the interface between the semiconductor selenium and the metal film, producing a photoelectric effect at the interface. The magnitude of the general potential difference is proportional to the illuminance of the light. The measurement range of the ultraviolet intensity meter is 0–15 mw/m^2^, and the measurement error is <5%.

### 2.5 Sensory evaluation

After drying, the sensory quality was assessed (according to the Chinese national standard of GB/T 23776‐2018) by a tasting panel of three panelists. Five quality aspects, including appearance, aroma, flavor, liquor color, and residue, were evaluated. All tasters had more than 5 years of experience in PT quality evaluation. A white matt evaluation table was used for sensory evaluation. Both the evaluation cup and bowl were made of white porcelain. The evaluation cup was cylindrical with a height of 75 mm and an outer diameter of 80 mm. The height of the evaluation bowl was 75 mm and the upper diameter was 80 mm. The 3‐g sun‐dried PT was weighed and infused in an evaluation cup with 250 ml freshly boiled water for 4 min. The liquor was then poured into the 440‐ml evaluation bowl. The residues were sniffed three times for aroma evaluation. Then, the liquor was first evaluated for intensity, clarity, and brightness. When the temperature dropped to 40℃, 5–10 ml liquor was drunk and swirled continuously by the tasters with their tongue tips. For the tea liquor taste, the aroma was expelled through the nose when the top of the tongue was swirling the liquor (Wang & Ruan, 2009). The evaluation of the tea leaf was then followed immediately. According to the weighting of each sensory attribute for green tea provided by the Chinese National Standard (GBT23776‐2018), the score of the overall quality was calculated by the following formula: appearance × 25% + aroma × 25% + flavor × 30% + liquor color × 10% + residue × 10%. The final scores were the mean values of the three experts. In this study, all PT samples were picked from similar tea trees and had the same tenderness, and hence, their appearance scores were 20 points from all the panelists.

### 2.6 Quantitative prediction models with image information

**FIGURE 2:**
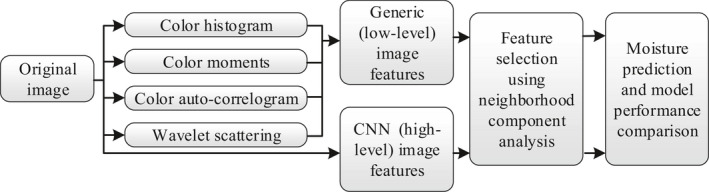
Flowchart of the moisture content prediction model

Different models were constructed to predict moisture content during the sun‐drying process and to predict the sensory scores after drying as well. As shown in Figure 2, the image information was used for moisture prediction first. The color histogram, color moments, color autocorrelogram, and wavelet scattering methods were used to extract low‐level image features of PT leaves, and a well‐trained Rank expansion network (RexNet; Han et al., 2020) was used as the CNN image extractor to extract high‐level image features. After neighborhood component analysis (NCA) feature selection, two predictors of MLP and GRU used generic features and CNN features as inputs to fit the moisture content and compare the prediction accuracy.

**FIGURE 3:**
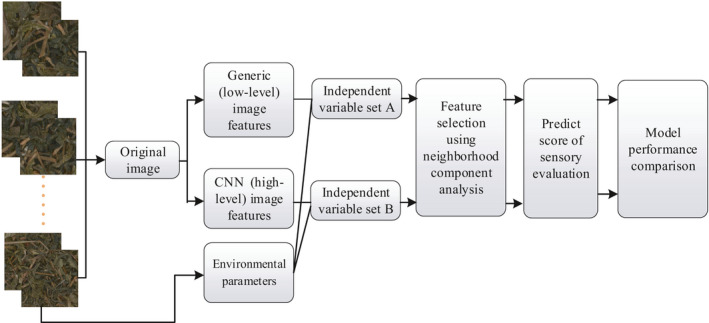
Flowchart of the sensory quality prediction model

As illustrated in Figure 3, both image information and environment parameters were input to a designed predictor for sensory score evaluation. After NCA feature selection, the *R*‐Square, RMSE, and RPD values of four models (low‐level image features + EP + MLP, low‐level image features + EP + GRU, high‐level image features + EP + MLP, and high‐level image feature + EP + GRU) were compared. Residual prediction deviation (RPD) was defined as the ratio of the standard deviation to the root mean square error (RMSE) in the prediction set (Liu et al., 2015). More accurate prediction models have larger *R*‐squared and RPD values, and smaller RMSE values. Based on RPD, prediction models are classified into three categories: Category A (RPD > 2), Category B (1.4 < RPD < 2), Category C (RPD < 1.4). Prediction models which successfully categorized A and B were presumed to have the potential to achieve satisfactory results (Chang et al., 2001).

#### 2.6.1 Wavelet scattering

**FIGURE 4:**
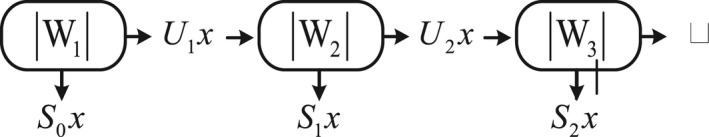
Wavelet scattering formed by wavelet modulus cascading

Wavelet scattering is a null‐parameter, handcrafted convolution network originally proposed by Mallat (2012) for generating stable‐invariant feature representation. Invariant image descriptors could be produced through sequential wavelet decomposition over multiple layers. The filters of the wavelet scattering network and CNN are different. The scattering network makes use of complex directional wavelet filters while CNN trains introduced convolution kernels for filtering. Invariant scattering coefficients *S*_m_ *x* and a subsequent layer of covariant wavelet module coefficients *U*_m+1_ *x* are the outcomes of |*W*_m_|. The average *S*_m_ *x* carries the low frequencies of *U*_m_ *x* while it loses all the high frequencies. |*W*_m_| transforms *U*_m_ *x* into the standard *S*_m_ *x* and a new layer *U*_m+1_ *x* of wavelet amplitude coefficients: |*W*_m_|(*U*_m_ *x*) = (*S*_m_ *x*, *U*_m+1_ *x*). For *m* = 0, *U*_0_ *x=x*, the scattering feature vector (*S_x_*) should be a concatenation of *S*_i_ *x* coefficients. A filter bank of low‐pass and high‐pass filters for implementing *W*_m_ operators is illustrated in Figure 4.

Compared with wavelet scattering, Fourier transform also can produce invariants, but its power spectrum depends on the second‐order moments (Bruna & Mallat, 2013). The information on higher order moments is collected by scattering transform, which improves discrimination for scattering representation. Consequently, employing scattering transform for feature learning has advantages in texture feature extraction.

#### 2.6.2 CNN image feature extractor

With the help of GPU, the training process of CNN can be effectively accelerated. In the case of a large amount of training data input, compared with the generic (low‐level) image feature extraction technique, CNN can extract high‐level features in the pictures and achieve higher accuracy. Consequently, CNN was used in this study as the feature extractor to compensate for the inefficiency and low accuracy of generic methods.

Convolution is a shift‐invariant operation, including the performance of locally weighted combinations across all the input images. The convolution layer is composed of several convolution kernels which are used to compute different feature maps. Depending on the set of chosen weights, various input features are revealed. Mathematically, the feature value at location (*i*, *j*) in the *k*‐th feature map of *l*‐th layer, $Z_{i,j,k}^{l}$ is calculated by,

(1) $$Z_{i,j,k}^{l}=W_{k}^{l\;T}X_{i,j}^{l}+b_{k}^{l}$$

where $W_{k}^{l}$ and $b_{k}^{l}$ are the weight vector and bias term, and $X_{i.j}^{l}$ is the input patch centered at location (*i*, *j*) of the *l*‐th layer. The weight of the kernel $W_{k}^{l}$ is shared by filters across the entire visual field, which can reduce the model complexity and make the CNN easier to be trained (Aloysius & Geetha, 2017).

In general, the traditional CNN consists of convolutional layers, activation layers, downsampling layers, fully connected layers (FC), and loss function. A FC is usually used to collect feature information extracted in the filtering stage. The earlier layers of a CNN tend to learn more low‐level elements such as edges and contours, which are then combined by the last layers to recognize complex high‐level image features of task‐specific objects (Dhillon & Verma, 2020). In short, by careful manipulation, an excellent CNN structure can effectively turn complex information into simple features.

**FIGURE 5:**
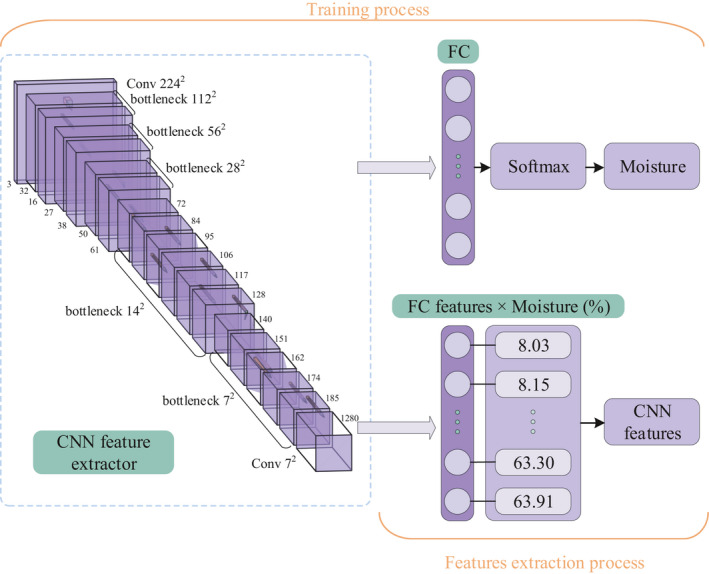
Flowchart of CNN image feature extraction model

In recent years, Mobilenet V2 has become one of the most popular CNN models that researchers and practitioners frequently use. It is based on an inverted residual structure, which provides more efficient and lightweight architecture. RexNet can be improved with Mobilenet V2. By making use of the representational bottleneck and squeeze‐and‐excitation attention module, it has achieved better model performance (Sandler et al., 2018). As shown in Figure 5, RexNet was used as a CNN image feature extractor in this study. Images of 546 samples and corresponding moisture content labels were input to the RexNet in the training process. PT images were augmented to increase the number of training data. The augmentation helped to improve the generalization of classifiers and reduce the possibility of overfitting. The augmentation methods included combinations of adding white noise, random reflection, random rotation, and random scaling in this study.

In the feature extraction process, the weights of the RexNet layers were frozen. All pictures were passed through the CNN feature extractor, and the FC's output vectors were obtained simultaneously. Finally, the multiplication of the FC feature vectors and 546 moisture content was used as the CNN image features. The expression is shown in (3):

(2) $${Z=YX}^{T}$$

Where ***Z*** is the CNN features of a single image (1 × 546), ***X*** is the fully connected vector (546 × 1), and ***Y*** is the 546 moisture content label vector (546 × 1).

#### 2.6.3 Neighborhood components analysis feature selection

Feature selection involves a selection of a small subset of original features by discarding redundant and inappropriate data. Reducing the dimensions of variables can increase the interpretability of the chosen features and reduce computing resources. As a nonparametric feature selection technique, NCA chooses features by measuring objective function, which calculates the regression loss over the training data (Goldberger et al., 2004). Feature selection can be achieved based on the significance of variable weights.

Given *n* observations:

(3) $$S=\left\{(x_{i},\;y_{i}),\;i=1,2,\ldots ,n\right\}$$

where *x_i_* are the feature vectors and *y_i_* are continuous response values. The aim is to predict the response of *y* given by the training set *S*.

Randomly pick a point Ref(*x*) from *S* as the reference point for *x*, and set the response value at *x* equal to the response value of the reference point Ref(*x*). The probability *P*(Ref(*x*)=*x_j_* |S) that point *x_j_* is picked from *S* as the reference point for *x* is expressed as:

(4) $$P\left(\text{Ref}\left(x\right)=x_{j}\text{|S}\right)=\frac{k(d_{w}\left(x,\;x_{j}\right))}{\sum_{j=1}^{n}k(d_{w}\left(x,\;x_{j}\right))}$$

Predicting response for *x_i_* using the data in *S^−i^*, the training set *S* excludes the point (*x_i_*, *y_i_*). The probability that point *x_j_* is picked as the reference point for *x_i_* is

(5) $$P_{\mathrm{ij}}=P\left(\text{Ref(}x)=x_{j}|S^{‐i}\right)=\frac{k(d_{w}\left(x,\;x_{j}\right))}{\sum_{j=1,j\neq i}^{n}k(d_{w}\left(x,\;x_{j}\right))}$$

Let *l* be a loss function that measures the difference between response value ${\hat{y}}_{i}$ and reference value *y_i_*. Then, the average value of $l(y_{i},\;{\hat{y}}_{i})$ is

(6) $$l_{i}=E\left(l\left(y_{i},\;{\hat{y}}_{i}\right)|S^{‐i}\right)=\sum_{j=1,\;j\neq 1}^{n}p_{\mathrm{ij}}l\left(y_{i},\;y_{j}\right)$$

The objective function for minimization is:

(7) $$f\left(w\right)=\frac{1}{n}\sum_{i=1}^{n}l_{i}+\lambda \sum_{r=1}^{p}W_{r}^{2}$$

The NCA method is used to select feature variables with higher weights as the input of the prediction model to improve the training speed of the model while ensuring prediction accuracy.

#### 2.6.4 MLP and GRU predictor

**FIGURE 6:**
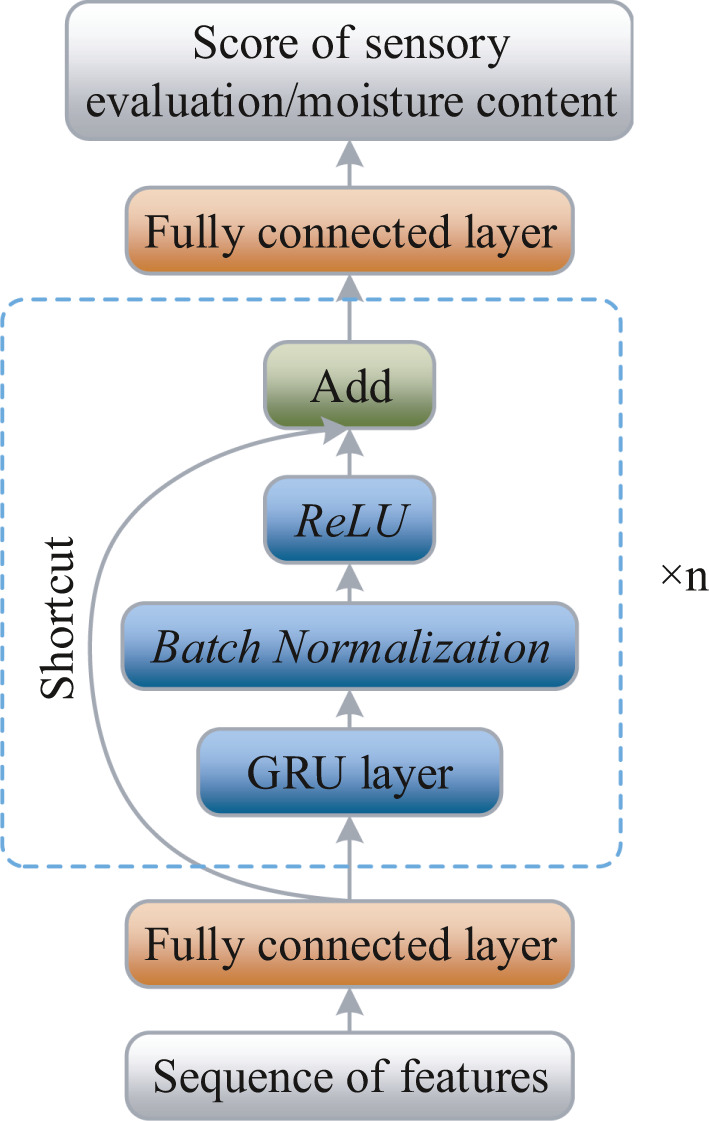
Proposed GRU architecture for prediction of moisture and sensory scores

The recurrent neural network (RNN) is an extension of a conventional feedforward neural network. However, RNN cannot avoid the gradient explosion problem (Yu et al., 2019), which limits its application. The long short‐term memory (LSTM) network not only uses a complex structure to overcome this challenge but it also causes slow computing speed. In contrast, a GRU network has only two gate structures, including an update gate and a reset gate. It can reduce computation as much as possible while solving the gradient explosion problems. At the same time, a multilayer GRU stacking could increase its prediction ability. In fact, in recent years, many methods have been reported to be used in deep CNNs, such as ReLU activation function, batch normalization layer, and shortcut connection (He et al., 2016). As illustrated in Figure 6, the above methods were used to deepen the GRU model further while obtaining a smaller loss value in this study.

To improve the prediction accuracy of GRU, the rectified linear unit (ReLU) was used to clip negative values to zero and keep the positive value unchanged (Hara et al., 2015). Activation functions can be used to combine the weighted sum of input and biases, in order to decide if a neuron could be fired. Therefore, the overall speed of neural network computation can be enhanced by avoiding computer exponentials and divisions: It simply outputs 0 when *X* < 0 and outputs a linear function when *X* ≥ 0, where *X* refers to the input vectors.

Multilayer CNNs are highly nonlinear as it is a cascade of several nonlinear operations. Therefore, Batchnorm was developed to improve the training process of neural networks by stabilizing the distributors of inputs (Ioffe & Szegedy, 2015), which plays an essential role in rectifying nonlinearity in CNN. It has been used in most DL models as a default setting. The shortcut connections were first used in residual network (He et al., 2016), which skips layers in the forward step of an input. This milestone architecture solves the problem that deep neural networks were slow in the training process and resulted in a better performance than similar counterparts.

As shown in Figure 3, generic (low‐level) and CNN (high‐level) image features were input into the GRU model, and the moisture content is fitted into the model in this study. The methods in Section 2.6.1 used low‐level color and texture features as input to the predictors, ignoring most high‐level image information, and resulting in model instability and poor generalization performance. On the other hand, using GRU predicted value instead of CNN labels as moisture prediction output has many advantages. The critical reason is GRU can infer values that do not exist in CNN labels based on the FC vectors, while CNN can only find specific labels as the model's output, which indirectly lowers the model performance. Hence, in this study, the combination of CNN image features and GRU predictor was proposed to predict moisture content and sensory cores of PT.

In summary, the above methods are organized as follows: (1) The HSV color histogram, L a* b* color moments, RGB color autocorrelogram, and wavelet scattering are used to extract the low‐level image features. (2) The RexNet CNN is used to extract the high‐level image features. (3) Low‐ and high‐level features are used to predict tea moisture during the sun‐drying process by MLP and GRU. (4) Low‐level features + EP and high‐level features + EP are used to predict sensory scores (including aroma, flavor, liquor color, residue, and total score) of sun‐dried tea by MLP and GRU. (5) All input variables of MLP and GRU predictor would be selected by the NCA method to reduce the number of variables.

## 3 RESULTS AND DISCUSSION

### 3.1 Moisture content prediction with image processing

Experiments were performed and moisture prediction models were established for the sun‐drying process. The low‐level image feature extractor (including HSV color histogram, L a* b* color moments, RGB color autocorrelogram, and wavelet scattering) and high‐level image feature extractor (CNN) were used to extract color and textural features of tea leaves. In order to simplify the modeling process and improve the model performance, the NCA feature selection method was applied to select crucial variables essential for moisture prediction. MLP and GRU were established as the moisture predictor.

#### 3.1.1 Variations of image features with moisture contents

**FIGURE 7:**
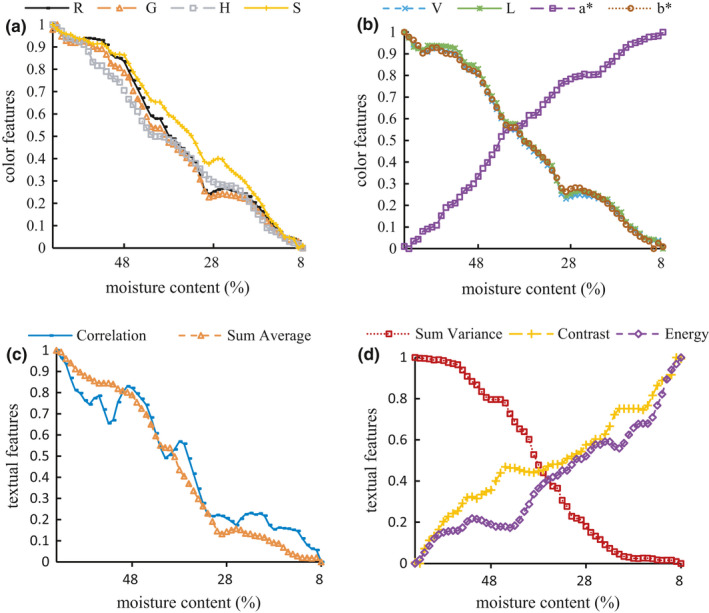
Changes in color (a and b) and texture (c and d) features with the variation of moisture contents

The trend curves of color features that varied with moisture contents are shown in Figure 7a,b. In the sun‐drying process, the tea leaves fade and color changed gradually. In order to extract color features, RGB images were converted into HSV and L a* b* color spaces. The (0, 1) normalization was conducted on the original data to investigate the dynamic principle of color and texture features. The coefficients between color features and moisture contents were obtained from the Spearman two‐tailed test (Zar, 1972). The coefficient of the blue channel's value is 0.094, which is not significant at the level of 0.05. The other coefficients are 0.724 for red channel (R), 0.750 for green channel (G), 0.703 for hue (H), 0.823 for saturation (S), 0.724 for value (V), 0.739 for lightness (L*), −0.670 for a* component (a*), and 0.815 for b* component (b*), which are significant at the level of 0.01.

During the sun‐drying process, the leaf texture also changed from spreading to shrinking form. The trend curves of texture features which varied with moisture contents are shown in Figure 7c,d. The texture features were extracted using the gray level co‐occurrence matrix (Haralick et al., 1973). The Spearman correlation coefficient of homogeneity is 0.095, which is not significant at the level of 0.05. The coefficients of contrast, energy, correlation, sum average, and sum variance are −0.354, −0.286, 0.558, 0.577, and 0.580, respectively, which are significant at the level of 0.01.

As shown in Figure 7, a* presented an upward trend with decreased moisture content, and R, G, H, S, V, L, b* demonstrated a declining linear trend. Contrast, energy, correlation, sum average, and sum variance of texture features presented a decreasing trend with reducing moisture content. Consequently, the color and texture features of PT leaves changed considerably with the loss of water during the sun‐drying process, which indicated that the moisture content of tea leaves could be predicted by color and textural features.

#### 3.1.2 Generic (low‐level) image feature extraction

**FIGURE 8:**
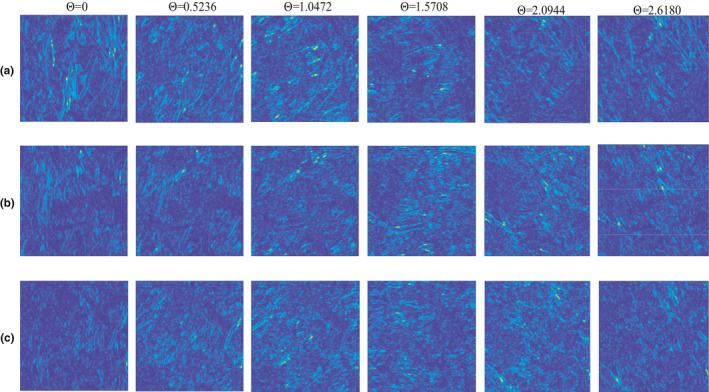
Wavelet scattering transform features of 63.91% (a), 29.96% (b), and 9.82% (c) moisture content with six different angels per wavelet

The extraction method of low‐level image features requires less computing resources, but it is easy to cause insufficient feature extraction and reduced prediction accuracy. To extract the generic (low‐level) features, the HSV color histogram, L a* b* color moments, and RGB color autocorrelogram of tea leaves’ images during the sun‐drying process were calculated. The low‐level image features were extracted by the following steps:

1. The hue, saturation, and value (HSV) histogram of tea leaf images was calculated. The photos were transformed into HSV color space with 8 × 2 × 2 equal bins. The HSV histogram feature includes a 1 × 32 vector, which was suggested by the literature (Barman & Choudhury, 2020).
2. Color moments is a practical, robust method to describe the image color features. The tea leaves' mean, standard deviation, skewness value of L, a*, b* channel were considered and formed a 1 × 9 vector, which was suggested by the same literature (Barman & Choudhury, 2020).
3. The color autocorrelogram was used to find the spatial correlation of the identical pixels. The input images were transformed into 64 colors in RGB as 4 × 4 × 4 color space, and calculated in a 1 × 64 color autocorrelogram feature with a distance set of 1,3,5,7, which was also suggested by the literature (Barman & Choudhury, 2020).
4. As illustrated in Figure 8, also referring to Section 2.6.1, the mean values along the second and third dimensions were calculated to obtain 391 element feature vectors for each image. This resulted in a significant reduction of data from 65,536 elements down to 391. The wavelet scattering method was applied for the extraction of textural features. As illustrated in Figure 7, the textural features extracted by wavelets with multiple angels have significant differences, allowing wavelet scattering to analyze tea leaf images’ texture features comprehensively.

#### 3.1.3 CNN (high‐level) image feature extraction

The extraction method of CNN image features has been improved with the development of GPU. With the assistance of GPU, compared with the low‐level image feature extraction methods, the training time of the CNN extractor has been shortened and the inference time of each picture has been reduced. The tea leaves with different moisture contents were photographed and the images were then used as the input of the CNN extractor to extract the CNN (high‐level) image features. The CNN extractor was mainly composed of a RexNet CNN.

As described in Section 2.6.2, the multiplication values of the FC's vector and moisture content were used as CNN features. The training options of CNN are shown in Table 1. Few training epochs have a poor classification effect during the training procedures, but more epochs lead to wastage of time. In this study, only part of the data were used for the determination of the suitable training epochs. After observation, it was found that 300 epochs can make RexNet achieve a stable accuracy. Consequently, 300 training epochs were selected under a comprehensive consideration. The 10‐fold cross‐validation method was applied to reflect the feature extraction capabilities of the RexNet mode. Finally, afterthe 10‐fold cross‐validation was performed, the accuracy of the training set was 99.45% and that of the validation set was 98.82%. However, the feature extraction process was only implemented on the validation set. In this study, all experiments were carried out under the PyTorch 1.4 framework with Ubuntu 18.04 operating system.

**TABLE 1:** CNN training options and parameters

CNN training options	CNN training parameters
Validation method	Tenfold cross‐validation
Optimizer	Stochastic gradient descent with the momentum of 0.9
Total training epochs	300 epochs
Mini batch size	64 images
Initial learning rate	0.01
Learning rate drop period	100 epochs
Learning rate drop factor	10%

In order to illustrate the essential information for the determination of the category, Grad‐CAMs and saliency maps were developed. Feature heat map pixels in the Grad‐CAMs were highlighted with a color gradient if they were considered critical for classification. Input image pixels in the saliency maps, on the other hand, were brightened based on the levels of significance to categories.

Grad‐CAM collected the global gradient to calculate the weight of the feature map. The weighted sum is obtained after including the weight of the category for all the feature maps. An image of a known classification was input into a trained CNN model in the process to show the saliency map. The derivatives of model output with respect to the input image's pixels were calculated using a guided backpropagation algorithm (Jin et al., 2020).

The feature maps in Table 2 show that the first convolutional layer focused on extracting edges of leaves and branches in tea leaf pictures. As shown in feature maps, branches and leaves are currently distinguished. The residues that cannot be used for classification are identified at the same time, which proves the advantages of CNN as an image feature extractor. As illustrated in Grad‐CAM and saliency map, the RexNet tended to analyze the leaf area changes caused by water loss when predicting the moisture contents of different tea leaves. As shown in Grad‐CAM (b) and saliency map (b), since the shape of the branches is not sensitive to changes in moisture content, water loss has less influence on the shape of branches than on the shape of leaves. Consequently, the branch area was blue in Grad‐CAM and black in the saliency map. On the other hand, the leaf area was red in Grad‐CAM and white in the saliency map, which proved that RexNet can be used to reflect the moisture content of tea leaf. In sample (c), the red area in Grad‐CAM was smaller than the white area in the saliency map and correct regions in the original picture; hence, the red area in Grad‐CAM (c) should be larger. As illustrated in the literature (Ju et al., 2021), the main reason for this phenomenon may be as follows: in Grad‐CAM, the heat map is generated by features from the shallow layers of CNN, which causes the presentation of high semantic features and loss of part of spatial information. This makes the final result area have a specific error, and an increase in training samples may help to solve this problem.

**TABLE 2:**
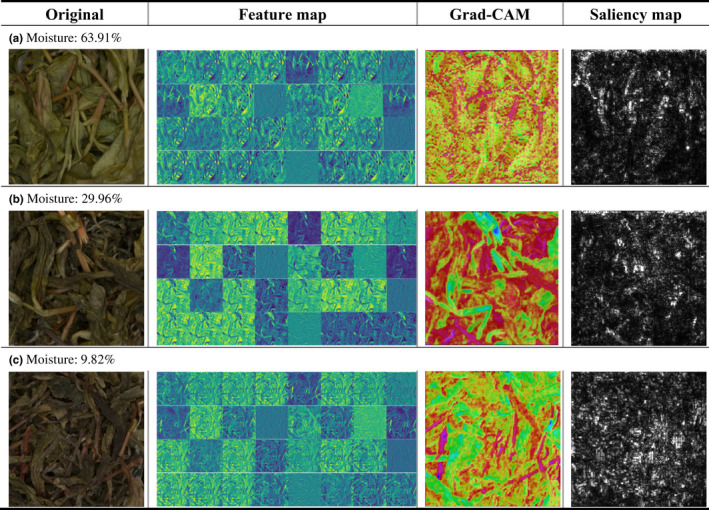
Feature map, Grad‐CAM, and saliency map of RexNet in different moisture contents

#### 3.1.4 Moisture content prediction based on MLP and GRU

In order to predict the moisture content of PT during the sun‐drying process, the extracted image information (including 688 variables for low‐level image features and 546 variables for high‐level features) was input into the MLP and GRU predictor. The image features contained collinearity, which caused inefficient modeling and more training time. Therefore, it is necessary to select crucial variables for prediction with NCA methods.

The results of the NCA feature selection are illustrated in Figure S1. The 10% features were selected from the original datasets. The numbers of image features were significantly decreased by 619 for low‐level image features and 490 for high‐level image features, suggesting the advantages of the NCA method. According to the results, color moments, color histogram, and wavelet scattering had a higher priority in predicting tea leaves’ moisture content. However, the CNN features of a sample with moisture content <10% and more than 60% were relatively hard to select, indicating that the CNN features with the tea moisture between 10% and 60% have a more significant impact on the accuracy of the moisture prediction.

Datasets selected by NCA were used to develop moisture content prediction models to accelerate the model's training speed and improve the model accuracy. The MLP is a multilayer feedforward network. It has a simple structure and can be trained quickly, but the prediction performance of MLP is usually worse than that of GRU. The data were divided into training and testing datasets using a random number generator to implement the 10‐fold cross‐validation in the neural network model. The size of hidden layers is 10. By using the optimized algorithm of the gradient descent method, the network was trained for 200 epochs to achieve a stable loss value and optimize the model performance. The same method used in Section 2.6.4 was also applied to determine the GRU training parameters. The training parameters of the GRU model are shown in Table 3.

**TABLE 3:** GRU training options and parameters

GRU training options	GRU training parameters
Validation method	Tenfold cross‐validation
Optimizer	Adaptive moment estimation
Total training epochs	300 epochs
Number of GRU hidden units	100
Number of shortcut block	2

Two image feature extractors (generic and CNN feature extractor) and two predictors (MLP and GRU) were combined to form four models. Only the image information was applied for moisture content prediction during the whole sun‐drying process. Moisture content prediction models were built in three situations, including the prediction for each batch of tea sample, tea sample in the same batch but at different sampling periods, and the overall samples. The *R*‐Square, RMSE, and RPD values of each model were applied to measure the prediction accuracy.

As illustrated in Figures S2 and Fig. S3, for moisture content prediction for each batch of tea, the prediction accuracy and stability of the four constructed models, which are denoted using RMSE values, decreased in the following order: 0.8529 of low‐level image feature + MLP, 0.6585 of low‐level image feature + GRU, 0.5635 of high‐level image feature + MLP, and 0.4332 of high‐level image feature + GRU. The *R* ^2^ values of the above models are .9988, .9992, .9994, and .9997, respectively. The RPD values of the above models are 25.9479, 34.1184, 40.2256, and 53.5894, respectively. Consequently, the point with the most significant prediction error in the CNN‐GRU model can still meet the relatively high moisture prediction accuracy for each batch of tea. The batch with the most significant error is shown in Figure S2, and the errors of other batches were smaller than the 74th batch.

As illustrated in Figures S4 and S5, for moisture prediction for tea at different sampling periods, there is no significant change in the ranking of model prediction performance compared with the prediction for each batch of tea. The average RMSE decreased in the following order: 0.7884 of low‐level image feature + MLP, 0.6338 of low‐level image feature + GRU, 0.5023 of high‐level image feature + MLP, and 0.2699 of high‐level image feature + GRU. The *R* ^2^ values of the above models are .9118, .9351, .9595, and .9882, respectively. The RPD values of the above models are 4.4482, 5.5428, 7.0313, and 13.1646, respectively. The sampling time of 4 h has a relatively larger prediction error and achieves satisfactory prediction accuracy (*R* ^2^ > .9 and RPD > 2). Therefore, the high‐level image features + GRU model is suitable for detecting various sampling times’ moisture contents.

For moisture content prediction for the overall samples, the data of the above two parts were merged and predictions were conducted. As shown in Figure S6 and Table 4, the combination of CNN image features and GRU predictor achieved the best performance, followed by high‐level image features + MLP, low‐level image features + GRU, and low‐level image features + MLP.

**TABLE 4:** Moisture detecting average accuracy of different models in each batch of tea, tea at various sampling times, and the overall tea samples

Samples	Evaluation methods	High‐level image features + GRU	High‐level image features + MLP	Low‐level image features + GRU	Low‐level image features + MLP
Each batch of tea	*R* ^2^	.9997	.9994	.9992	.9988
	RMSE	0.4332	0.5635	0.6585	0.8529
	RPD	53.5894	40.2256	34.1184	25.9479
Various sampling times	*R* ^2^	.9882	.9595	.9351	.9118
	RMSE	0.2699	0.5023	0.6338	0.7884
	RPD	13.1646	7.0313	5.5428	4.4482
All tea samples	*R* ^2^	.9986	.9981	.9977	.9958
	RMSE	0.7508	0.8621	0.9533	1.2856
	RPD	26.3513	22.9500	20.7544	15.3898

In summary, as illustrated in Table 4, although the values of RMSE are different in the above three cases of moisture content prediction, the RMSE rankings of the four models were consistent. The values of *R* ^2^ are >.9, and RPD values are >2. Consequently, the CNN‐GRU model can achieve good prediction accuracy under various conditions.

### 3.2 Sensory quality evaluation of sun‐dried PT

Experimental data were selected and analyzed to build more effective prediction models for sensory quality prediction. As reported in the literature (Chan et al., 2009; Roshanak et al., 2016), during the sun drying of PT, the chemical substances’ changes are closely related to the final sensory quality, and moisture content prediction models are helpful in the evaluation of the sensory scores. The image features (reflecting moisture content) and the EP were input into the MLP and GRU prediction models. The results of RMSE were then compared for the selection of the most effective combination to reduce the total input parameters, improve model accuracy, and find the most critical EP that affect the sensory quality mostly.

#### 3.2.1 Correlation between image information, EP, and sensory scores

As mentioned in Section 3.1.1, the correlation coefficients between image information, EP, and sensory scores were calculated with the Spearman two‐tailed test. The larger the correlation coefficient is, the brighter the grid appears. The EP used here are the average values of the whole sun‐drying process.

**FIGURE 9:**
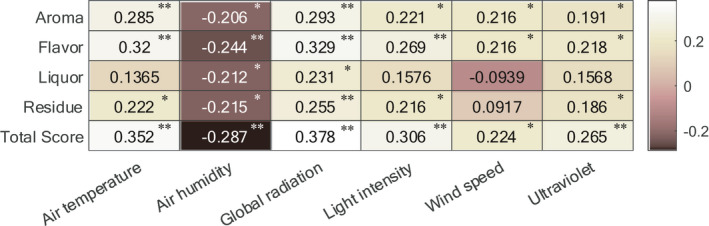
The Spearman correlation between sensory scores and environmental parameters (* and ** represent significance level of 0.05 and 0.01, respectively)

**FIGURE 10:**
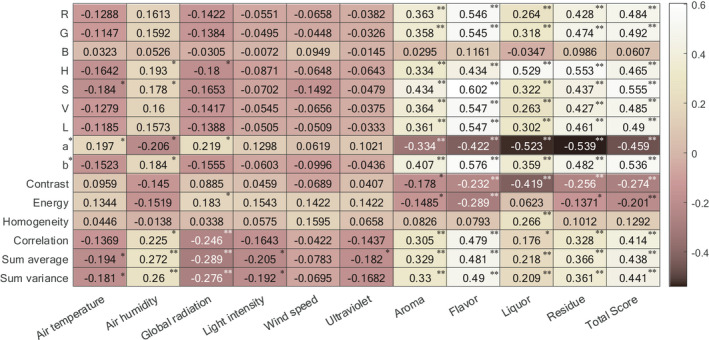
The Spearman correlation between image information and environmental parameters, image information and sensory scores (* and ** represent significance level of 0.05 and 0.01, respectively)

As illustrated in Figure 9, air humidity is significantly but negatively correlated with sensory scores. Air temperature, global radiation, light intensity, wind speed, and ultraviolet significantly are positively correlated with sensory scores. As illustrated in Figure 10, a*, contrast, and energy are significantly negatively correlated with sensory scores. The remaining image features are positively correlated with sensory scores, in which the correlation coefficient of B and homogeneity are not significant, and R, G, H, S, V, L, b*, correlation, sum average, and sum variance are significant. Most image features are not significantly correlated with EP. Besides air humidity, most of the EP are positively correlated with image features.

The reasons for the above phenomena may be as follows: (1) too high air humidity reduced the quality of PT in the sun‐drying process, and a reasonable increase in air temperature, global radiation, light intensity, wind speed, and ultraviolet helped to improve sensory scores. (2) Image features and EP can reflect the changes in sensory scores. (3) The correlations between EP and image features are not significant. The reason might be that the image features were affected by multiple EP in the sun‐drying process, so a single environmental parameter could not fully reflect the changes in image features.

#### 3.2.2 Image features’ extraction and sensory scores' prediction

In order to fit the sensory scores of the sun‐dried PT, both EP and image information were used to evaluate the aroma, flavor, liquor color, residue, and total score of sun‐dried PT. After adding various combinations of EP to the models, the significance of EP was measured. By comparing the change rate of RMSE, the most compelling environmental parameter combination is proposed, which uses fewer inputs to achieve higher accuracy.

The CNN‐GRU model with the highest accuracy had been used in moisture content prediction to evaluate the sensory scores but did not incorporate EP into the model. As illustrated in Figure S7, sensory scores cannot be evaluated when the image information only was input into the GRU model. Subsequently, EP (including air humidity, air temperature, global radiation, light intensity, wind speed, and ultraviolet radiation) were used as GRU input variables. The NCA feature selection method was used to decrease the number of inputs and improve the model performance.

**FIGURE 11:**
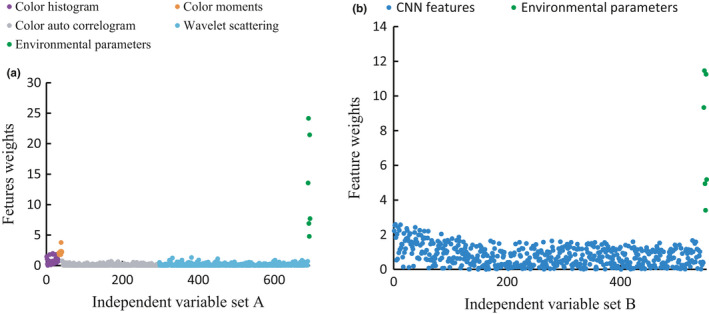
NCA feature weights of independent variable set A (a) and independent variable set B (b)

As illustrated in Figure 11a,b, with EP' join, the NCA weights of air temperature, air humidity, light intensity, global radiation, wind speed, and ultraviolet were higher than any single image feature, which further emphasized the significance of EP in PT sensory quality prediction.

Furthermore, as illustrated in Figure S8, combinations of different EP were separately input into the CNN‐GRU model to attempt the prediction possibility. As shown in Figures S9–S11, the prediction performance of the four models was different in terms of sensory score prediction. With regard to aroma, flavor, liquor color, residue, and total score, the CNN‐GRU model obtained the best prediction performance compared with other models. The accuracy of the CNN‐GRU model for sensory evaluation, which was denoted by RMSE, decreased in the following order: 0.3135 for the total score, >0.2656 for flavor, >0.2005 for aroma, >0.1521 for liquor color, >0.1332 for residue. The *R* ^2^ values of the above sensory scores are .6900, .8777, .9119, .9380, and .9433, respectively. The RPD values of the above sensory scores are 1.8829, 3.3682, 4.1225, 4.4098, and 4.5940, respectively.

As illustrated in Figures S9–S11, for evaluation of aroma, flavor, liquor color, residue, and total score, there existed the most significant error when only image features were used as input. Overall, as illustrated in Table 5, the average *R*‐Square, RMSE, and RPD values of the models from low to high were ranked as follows: high‐level image feature + EP + GRU, low‐level image features + EP + GRU, high‐level image features + EP + MLP, and low‐level image features + EP + MLP. The above sequence is different from the ranking of moisture content prediction. The possible reason is as follows: when predicting the moisture content, the color and texture of images were closely related to water, and the degree of image information extraction has a more significant impact on the prediction performance of models. Therefore, whether to use CNN as an image feature extractor is a crucial factor. In contrast, when evaluating sensory scores, EP are in a more critical position compared to image information, so the advantages of GRU were more easily reflected. However, in all cases, the CNN‐GRU model achieved the highest prediction accuracy, which fully proved the advancement and superiority of the deep learning‐based moisture content prediction and sensory quality determination model.

**TABLE 5:** Sensory quality evaluation average accuracy of different models using various environment parameters as inputs

Samples	Evaluation methods	High‐level image features + GRU	Low‐level image features + GRU	High‐level image features + MLP	Low‐level image features + MLP
Aroma	*R*‐Square	.9119	.6880	.6370	.4848
	RMSE	0.4897	1.1440	1.2812	1.7458
	RPD	4.1225	1.5146	1.3429	0.9669
Flavor	*R* ^2^	.8777	.7303	.6008	.4757
	RMSE	0.6699	1.1288	1.5250	1.9342
	RPD	3.3682	1.8130	1.2490	0.9796
Liquor color	*R* ^2^	.9380	.8675	.7895	.7027
	RMSE	0.2562	0.4046	0.5363	0.6750
	RPD	4.4098	2.7647	1.9661	1.5632
Residue	*R* ^2^	.9433	.8698	.8064	.7567
	RMSE	0.2302	0.3735	0.4735	0.5503
	RPD	4.5940	2.6837	2.0669	1.7700
Total score	*R* ^2^	.6900	.4167	.2423	.1697
	RMSE	1.1578	2.0739	3.0444	3.7053
	RPD	1.8829	0.8480	0.5473	0.4389

From Figures S9–S11, it can also be found that an increase in the number of EP does not necessarily improve the prediction accuracy of the sensory score of the CNN‐GRU model. When predicting the sensory scores, the accuracy of the model prediction is ranked the same. For aroma prediction, the average RMSE of the four models decreased as follows: 1.7458 of low‐level image features + EP + MLP > 1.2812 of high‐level image features + EP + MLP > 1.1440 of low‐level image features + EP + GRU > 0.4897 of high‐level image feature + EP + GRU. The *R* ^2^ values of the above models are .4848, .6370, .6880, and .9119, respectively. The RPD values of the above models are 0.9669, 1.3429, 1.5146, and 4.1225, respectively. For flavor prediction, the RMSE values of the four models are: 1.9342, 1.5250, 1.1288, 0.6699 (*R* ^2^: .4757, .6008, .7303, .8777, RPD: 0.9796, 1.249, 1.813, 3.3682). For liquor color prediction, the RMSE values of the four models are: 0.6750, 0.5363, 0.4046, 0.2562 (*R* ^2^: .7027, .7895, .8675, .938, RPD: 1.5632, 1.9661, 2.7647, 4.4098). For residue prediction, the RMSE values of the four models are: 0.5503, 0.4735, 0.3735, 0.2302 (*R* ^2^: .7567, .8064, .8698, 0.9433, RPD: 1.77, 2.0669, 2.6837, 4.594). For total score prediction, the RMSE values of the four models are: 3.7053, 3.0444, 2.0739, 1.1578 (*R* ^2^: .1697, .2423, .4167, .69, RPD: 0.4389, 0.5473, 0.848, 1.8829).

In order to achieve a high prediction accuracy and minimize the number of required EP, the highest accuracy points of different numbers of EP are analyzed and shown in Figures S9–S11 and Table 5. For example, when only three EP were input to the model, for evaluation of aroma and flavor, the CNN‐GRU model using air temperature + air humidity + light intensity as the EP got the local minimum RMSE value. For liquor color, residue, and total score, the best is air temperature + wind speed + ultraviolet.

**FIGURE 12:**
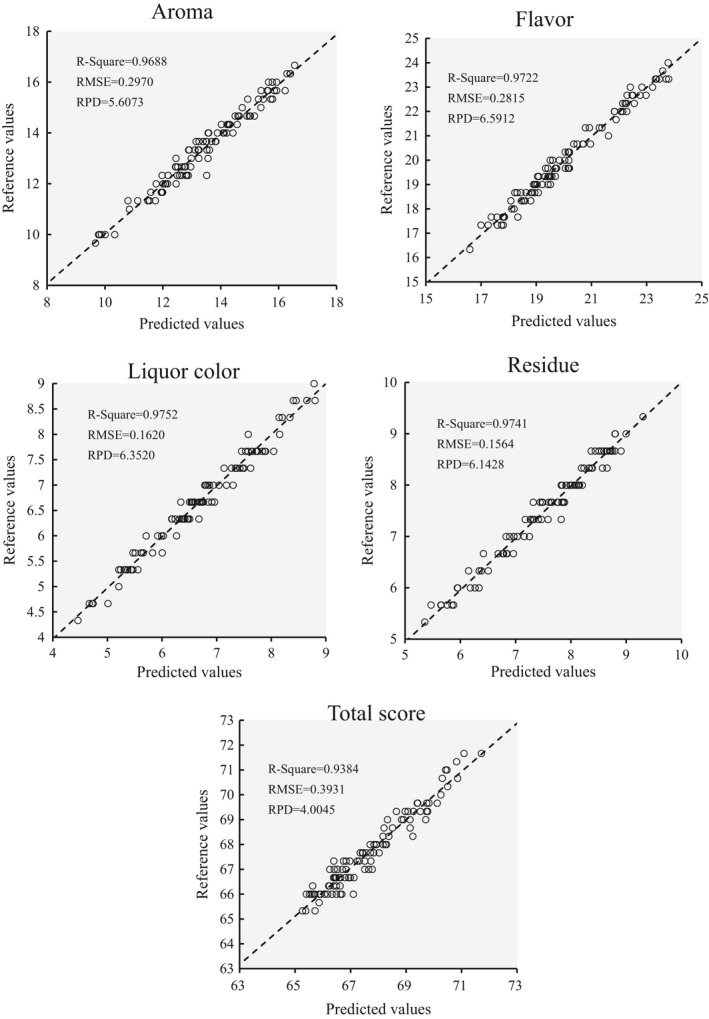
Scattergram of sensory score evaluation reference and predicted values using recommended environment parameters as CNN‐GRU model inputs

The RMSE value and change rate of the points in Tables S1–S3 are shown in Table 6. As illustrated in Table 7, the points with the fastest RMSE reduction were selected as the most efficient combination of EP. The combinations in Table 7 were used to evaluate the sensory scores of PT after the sun‐drying process, and the scattergram results are shown in Figure 12. It is evident that usage of the recommended environment parameters can accurately evaluate the aroma, flavor, liquor color, residue, and total score of PT. This conclusion is important for the optimization of the sun‐drying process and improvement of the sensory quality of PT.

**TABLE 6:** Max *R*‐Square (min RMSE, max RPD) point using a different number of environmental parameters as inputs

	Single environmental parameter	Two environmental parameters	Three environmental parameters	Four environmental parameters	Five environmental parameters	Highest accuracy environmental parameters
Aroma	Ultraviolet	Air humidity + Light intensity	Air temperature + Air humidity + Light intensity	Air temperature + Light intensity + Wind speed + Ultraviolet	Air temperature + Air humidity + Global radiation + Wind speed + Ultraviolet	Air temperature + Air humidity + Global radiation + Light intensity + Wind speed + Ultraviolet
Flavor			Air temperature + Wind speed + Ultraviolet			
Liquor color						
Residue						
Total score						

**TABLE 7:** Recommended environment parameters for sensory quality determination

Sensory evaluation index	Selected environmental parameters
Aroma	Air humidity + Light intensity
Flavor	Air temperature + Wind speed + Ultraviolet
Liquor color	Air temperature + Wind speed + Ultraviolet
Residue	Air humidity + Light intensity
Total score	Air temperature + Wind speed + Ultraviolet

## 4 CONCLUSION AND FUTURE WORKS

1. The EP and moisture contents (reflected through image information) are essential attributes for the evaluation of the sensory quality of PT during the sun‐drying process. Compared with other literature, this work is the first to use image information and EP to predict moisture contents (including the prediction for each batch of tea, tea at different sampling periods, and the overall samples) and the sensory quality of PT in a fast speed.
2. In this work, an ultraportable and low‐cost industrial camera and a meteorological monitoring device were used to collect data during the sun‐drying process. Moreover, a deep‐learning‐based high‐level image feature extractor and sensory score predictor were developed, which achieved high accuracy (RMSE values of 0.297 vs. 1.3311 for aroma, 0.2815 vs. 1.4976 for flavor, 0.162 vs. 0.4777 for liquor color, 0.1574 vs. 0.4701 for residue, and 0.3931 vs. 2.5318 for total score; *R* ^2^ values of .4805 vs. .9688, .4875 vs. .9772, .7541 vs. .9752, .7904 vs. .9741, 0.0039 vs. 0.8906; RPD values of 1.1365 vs. 5.6073, 0.9893 vs. 6.5912, 1.6696 vs. 6.352, 1.8232 vs. 6.1428, 0.2855 vs. 4.0045).
3. By comparing all the RMSE values of different EP, the crucial EP that should be firstly measured and regulated are suggested as follows: air humidity + light intensity for aroma and residue; air temperature + wind speed + ultraviolet for flavor, liquor color, and total score. These promising results provided suitable and accurate models for the optimization of the sensory quality prediction by regulating the EP. This convenient tool is also useful for large‐scale commodity production lines.

Compared with the traditional models with low‐level image feature extractors and MLP accessories, the deep learning‐based models exhibited good performance by using the features selected by the NCA method. The proposed prediction models have gotten satisfactory results: in moisture content prediction during sun drying with RMSE values of 0.4332 for each batch of tea, 0.2699 for tea at different sampling periods, and 0.7508 for the overall samples. The *R* ^2^ values of the above samples are .9997, .9882, and .9986, respectively. The RPD values of the above samples are 53.5894, 13.1646, and 26.3513, respectively. This study proved that the designed system can be used to evaluate the sensory scores of PT accurately. Moreover, the proposed combinations of different EP can also provide a valuable reference in the development of a new sun‐drying system.

Future works are recommended to optimize the PT sun‐drying procedure by changing part of the EP only. Efforts should be made to improve the sensory quality and insure the consistency of the sensory scores of different batches of tea products.

## Supporting information

Supplementary Material
