# Supplementary material for: Moisture contents and product quality prediction of Pu‐erh tea in sun‐drying process with image information and environmental parameters
Source: Food Sci Nutr. 2022 Feb 22;10(4):1021–38. doi: 10.1002/fsn3.2699 (PMC9007301; doi:10.1002/fsn3.2699)
Supplement: Supplementary file 1 — Supplementary Material [file FSN3-10-1021-s001.docx]

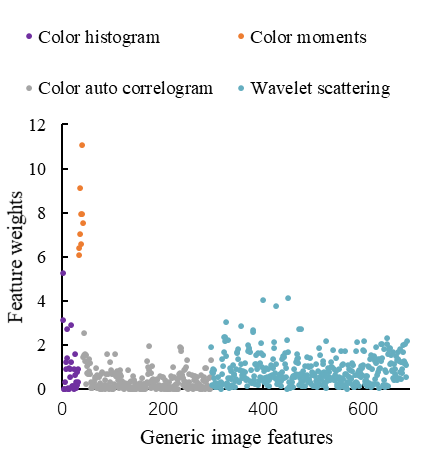


(a)


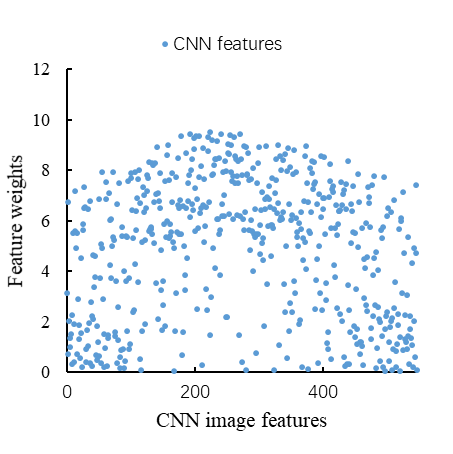


(b)

**Figure S1.** NCA feature weights of Generic (a) and CNN image feature extractor (b)

**Figure S2.** Scattergram of 74^th^ batch of tea's reference and predicted moisture contents using CNN-GRU model

(a)

(c)

(b)

**Figure S3.** R-Square (a), RMSE (b) and RPD (c) of different models in detecting 100 batches’ moisture contents

(
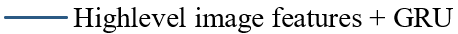

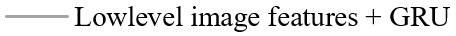

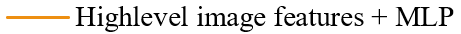

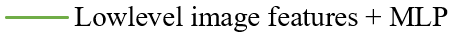
)

**Figure S4.** Scattergram of four hours’ sun-drying periods’ reference and predicted moisture contents using CNN-GRU model

(a)

(b)

(c)

**Figure S5.** R-Square (a), RMSE (b) and RPD (c) of different models in detecting various sampling times' moisture contents

(
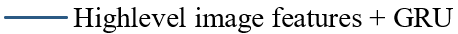

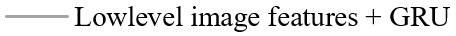

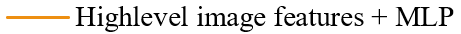

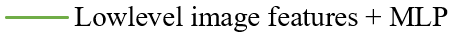
)

**Figure S6.** Scattergram of all tea samples’ reference and predicted moisture contents using High-level image features + GRU model

**Figure S7.** Scattergram of sensory score evaluation's reference and predicted values without using environmental parameters as CNN-GRU model inputs

**Figure S8.** Schematic diagram of various environmental parameters combination in prediction of sensory scores


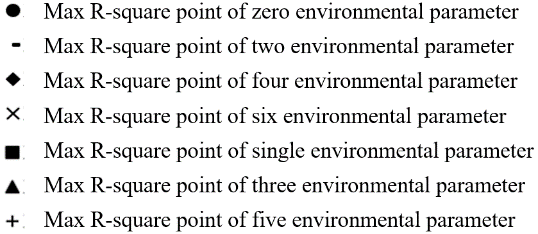


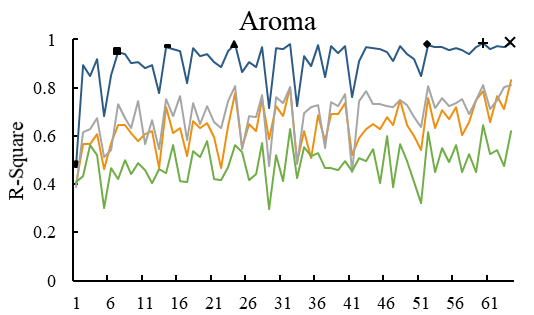

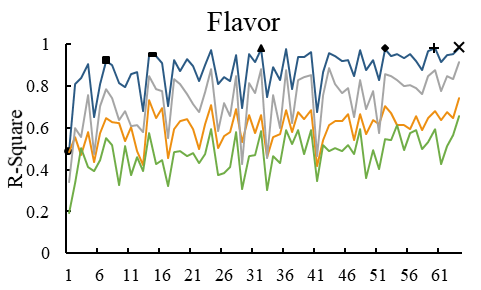

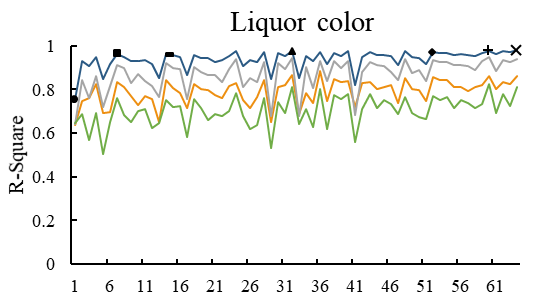

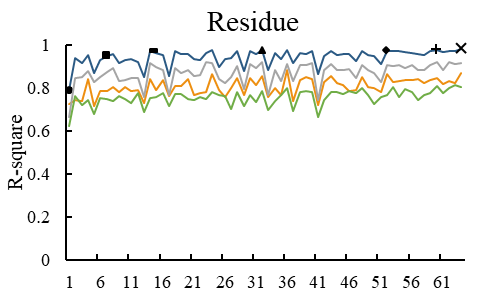

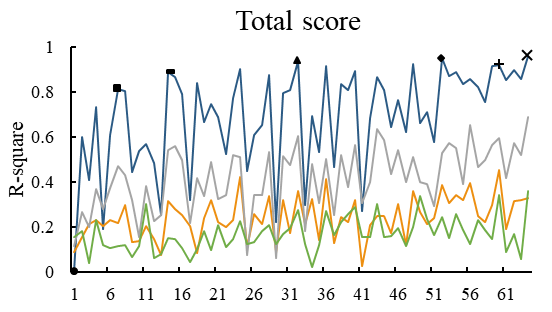

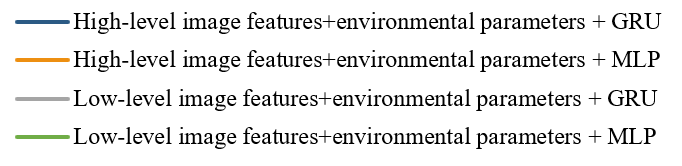


**Figure S9.** R-Square of different models using various environment parameters as inputs


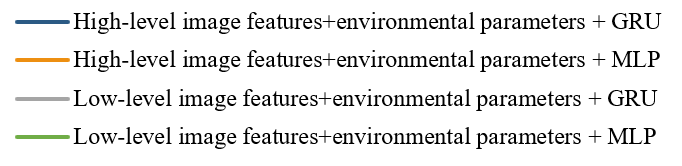


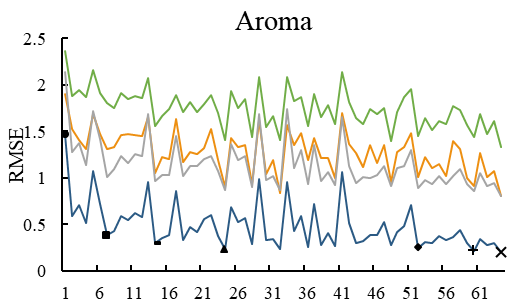

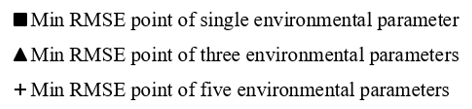

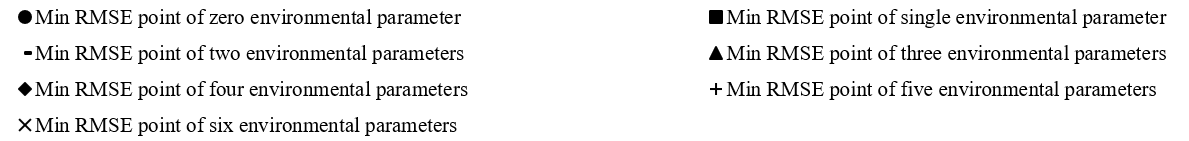

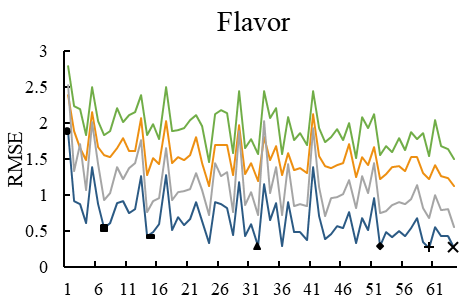

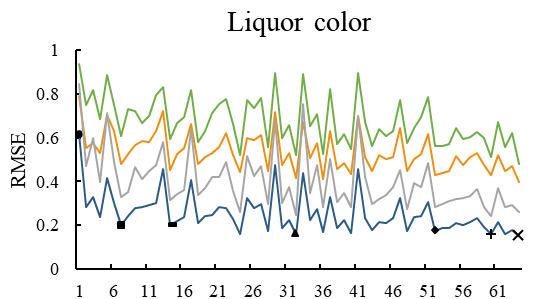

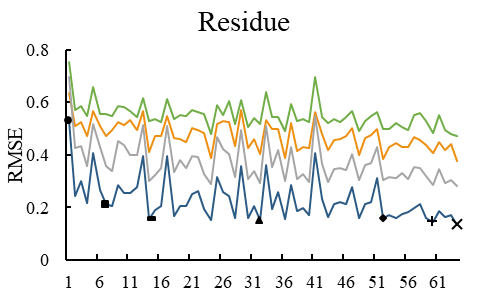

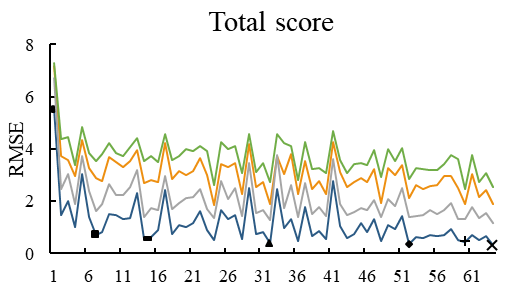


**Figure S10.** RMSE of different models using various environment parameters as inputs


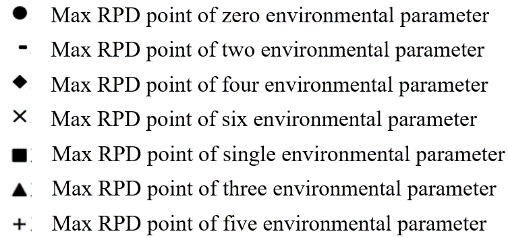

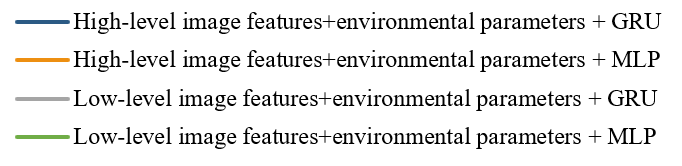


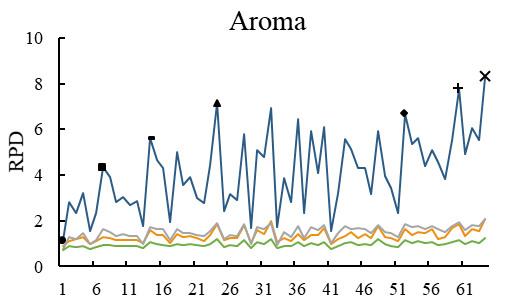

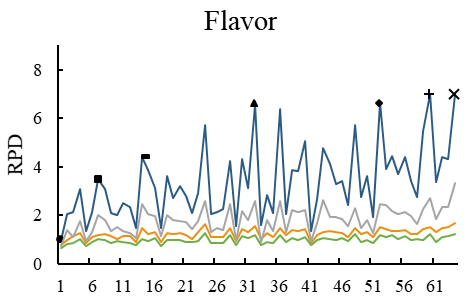

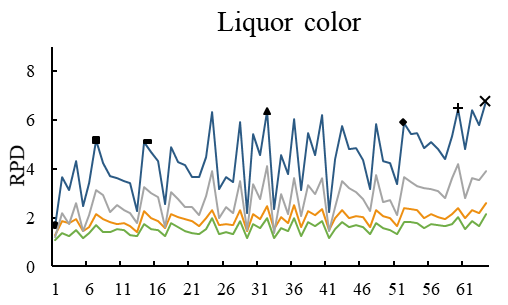

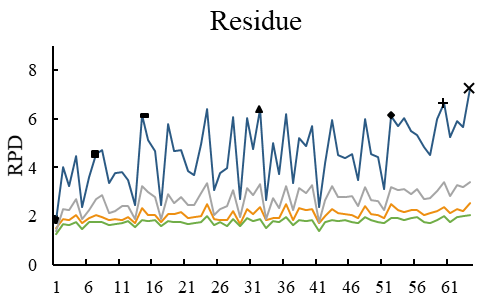

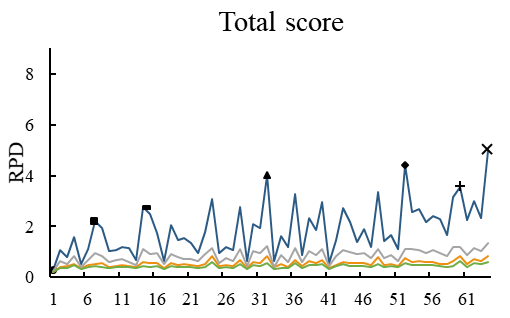


**Figure S11.** RPD of different models using various environment parameters as inputs

**Table S1** R-Square change rate of different number of environmental parameters

| Number of environmental parameters | Aroma R-Square | Rate of change | Flavor R-Square | Rate of change | Liquor color R-Square | Rate of change | Residue R-Square | Rate of change | Total score R-Square | Rate of change |
| --- | --- | --- | --- | --- | --- | --- | --- | --- | --- | --- |
| 1 | 0.9484 | - | 0.9197 | - | 0.9624 | - | 0.9512 | - | 0.8148 | - |
| 2 | 0.9688 | **2.15%** | 0.9481 | 3.09% | 0.9611 | -0.13% | 0.9741 | **2.41%** | 0.8906 | **9.30%** |
| 3 | 0.9804 | 1.20% | 0.9772 | **3.08%** | 0.9752 | **1.47%** | 0.9754 | 0.13% | 0.9384 | 5.38% |
| 4 | 0.9774 | -0.30% | 0.9773 | 0.00% | 0.9711 | -0.42% | 0.9733 | -0.21% | 0.9491 | 1.13% |
| 5 | 0.9836 | 0.63% | 0.9796 | 0.24% | 0.9761 | 0.51% | 0.9780 | 0.48% | 0.9218 | -2.87% |
| 6 | 0.9855 | 0.20% | 0.9807 | 0.11% | 0.9784 | 0.23% | 0.9819 | 0.40% | 0.9601 | 4.15% |

**Table S2** RMSE change rate of different number of environmental parameters

| Number of environmental parameters | Aroma RMSE | Rate of change | Flavor RMSE | Rate of change | Liquor color RMSE | Rate of change | Residue RMSE | Rate of change | Total score RMSE | Rate of change |
| --- | --- | --- | --- | --- | --- | --- | --- | --- | --- | --- |
| 1 | 0.3833 | - | 0.5328 | - | 0.1996 | - | 0.2125 | - | 0.7126 | - |
| 2 | 0.2970 | **-22.52%** | 0.4212 | -20.94% | 0.2020 | 1.21% | 0.1574 | **-25.93%** | 0.5698 | -20.03% |
| 3 | 0.2333 | -21.44% | 0.2815 | **-33.16%** | 0.1620 | **-19.79%** | 0.1512 | -3.91% | 0.3931 | **-31.01%** |
| 4 | 0.2496 | 7.00% | 0.2805 | -0.36% | 0.1747 | 7.83% | 0.1581 | 4.50% | 0.3573 | -9.12% |
| 5 | 0.2145 | -14.06% | 0.2657 | -5.29% | 0.1595 | -8.74% | 0.1458 | -7.78% | 0.4425 | 23.86% |
| 6 | 0.2005 | -6.56% | 0.2656 | -0.05% | 0.1521 | -4.64% | 0.1332 | -8.64% | 0.3135 | -29.15% |

**Table S3** RPD change rate of different number of environmental parameters

| Number of environmental parameters | Aroma RPD | Rate of change | Flavor RPD | Rate of change | Liquor color RPD | Rate of change | Residue RPD | Rate of change | Total score RPD | Rate of change |
| --- | --- | --- | --- | --- | --- | --- | --- | --- | --- | --- |
| 1 | 4.3446 | - | 3.4827 | - | 5.1565 | - | 4.5501 | - | 2.2091 | - |
| 2 | 5.6073 | **29.06%** | 4.4053 | 26.49% | 5.0948 | -1.20% | 6.1428 | **35.00%** | 2.7626 | 25.05% |
| 3 | 7.1377 | 27.29% | 6.5912 | **49.62%** | 6.3520 | **24.68%** | 6.3926 | 4.07% | 4.0045 | **44.96%** |
| 4 | 6.6709 | -6.54% | 6.6148 | 0.36% | 5.8905 | -7.27% | 6.1172 | -4.31% | 4.4062 | 10.03% |
| 5 | 7.7620 | 16.36% | 6.9842 | 5.58% | 6.4543 | 9.57% | 6.6334 | 8.44% | 3.5575 | -19.26% |
| 6 | 8.3068 | 7.02% | 6.9873 | 0.05% | 6.7682 | 4.86% | 7.2605 | 9.45% | 5.0209 | 41.14% |
